# A prenatal case with discrepant findings between non-invasive prenatal testing and fetal genetic testings

**DOI:** 10.1186/1755-8166-7-48

**Published:** 2014-07-16

**Authors:** Qiong Pan, Baojuan Sun, Xiaoli Huang, Xin Jing, Hailiang Liu, Fuman Jiang, Jie Zhou, Mengmeng Lin, Hongni Yue, Ping Hu, Ying Ning

**Affiliations:** 1 Laboratory of Clinical Genetics, Department of Prenatal Diagnosis, Huaian Maternal and Child Health Care Hospital of Jiangsu Province, Huaian 223002, China; 2 State key Laboratory of Reproductive Medicine, Department of Prenatal Diagnosis, Nanjing Maternity and Child Health Care Hospital Affiliated to Nanjing Medical University, 123 Tianfei Street, Nanjing 210029, China; 3 iGenomics, Guangzhou, China; 4 BGI-Shenzhen, Shenzhen, China

**Keywords:** Non-invasive prenatal testing, Massively parallel sequencing, Mosaicism

## Abstract

At 17^+4^ week, non-invasive prenatal testing (NIPT) results of a 24-years-old mother showed high risk of monosomy X (45, X). Abnormally shaped head and cardiac defects were observed in prenatal ultrasound scan at 19^+3^ week. Amniocentesis conducted at 19^+3^ week identified karyotype 47, XX, +18, which suggested that the NIPT failed to detect trisomy 18 (T18) in this case. With a further massively parallel sequencing (MPS) of maternal blood, fetal and placental tissues, we found a confined placental mosaicism (CPM) with non-mosaic T18 fetus and multiclonal placenta with high prevalence of 45, X and low level of T18 cells. FISH and SNP-array evidence from the placental tissue confirmed genetic discrepancy between the fetus and placenta. Because the primary source of the fetal cell-free DNA that NIPT assesses is mostly originated from trophoblast cells, the level of T18 placental mosaicism may cause false negative NIPT result in this rare case of double aneuploidy.

## Background

Non-invasive prenatal testing for common fetal aneuploidies, in particular trisomy 21 and 18, by massively parallel sequencing (MPS) of maternal plasma DNA is an extremely efficient screening test with sensitivity and specificity of over 99% [1].

## **Case** presentation

Here we report a rare case with mosaic monosomy X and trisomy 18 in placenta, which induced a false negative NIPT result. Results of the 2nd trimester combined test (AFP 0.65 MoM and hCGb 4.32 MoM) indicated that the pregnancy of the 24-year-old mother (“gravida 2, para 0”, G2P0) was at high risk of Down syndrome (1/45). As the following screening, a NIPT test was then performed at 17^+4^ week of gestation by maternal peripheral blood collection, cell-free DNA (cfDNA) extraction, library construction and sequencing through Illumina HiSeq2000 platform [2].

**Figure 1:**
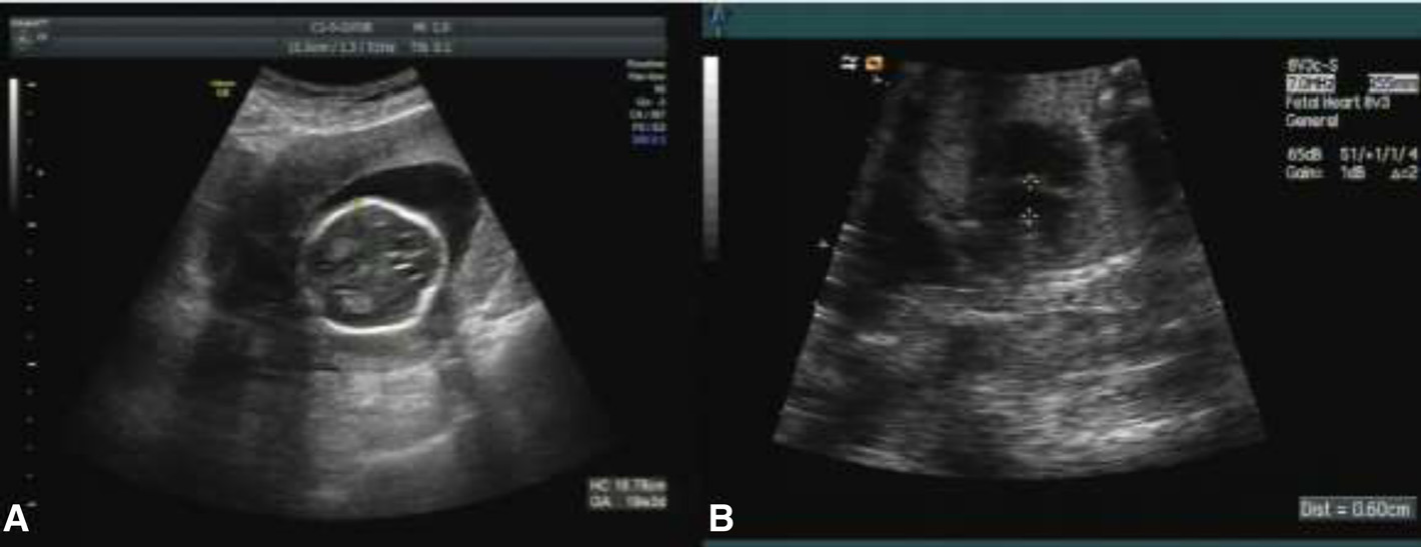
**Abnormal sonographic features of fetus at 19th week. A**. strawberry-shaped head. **B**. Four-chamber view of the fetal heart showing a ventricular septal defect (0.6 cm diameter).

**Figure 2:**
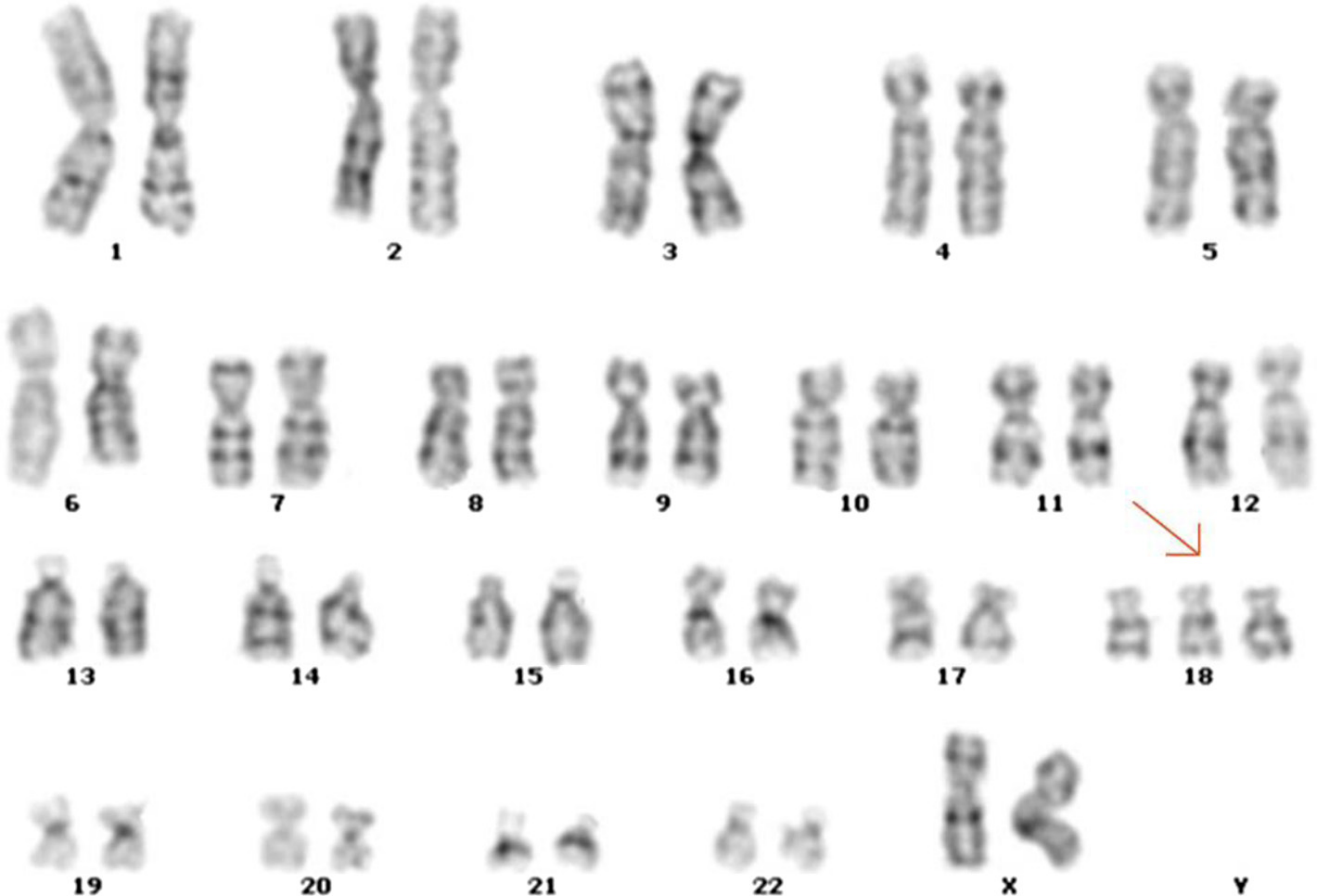
Conventional karyotype analysis of cultured amniocytes shows the fetal karyotype as 47, XX, +18 (trisomy 18).

**Figure 3:**
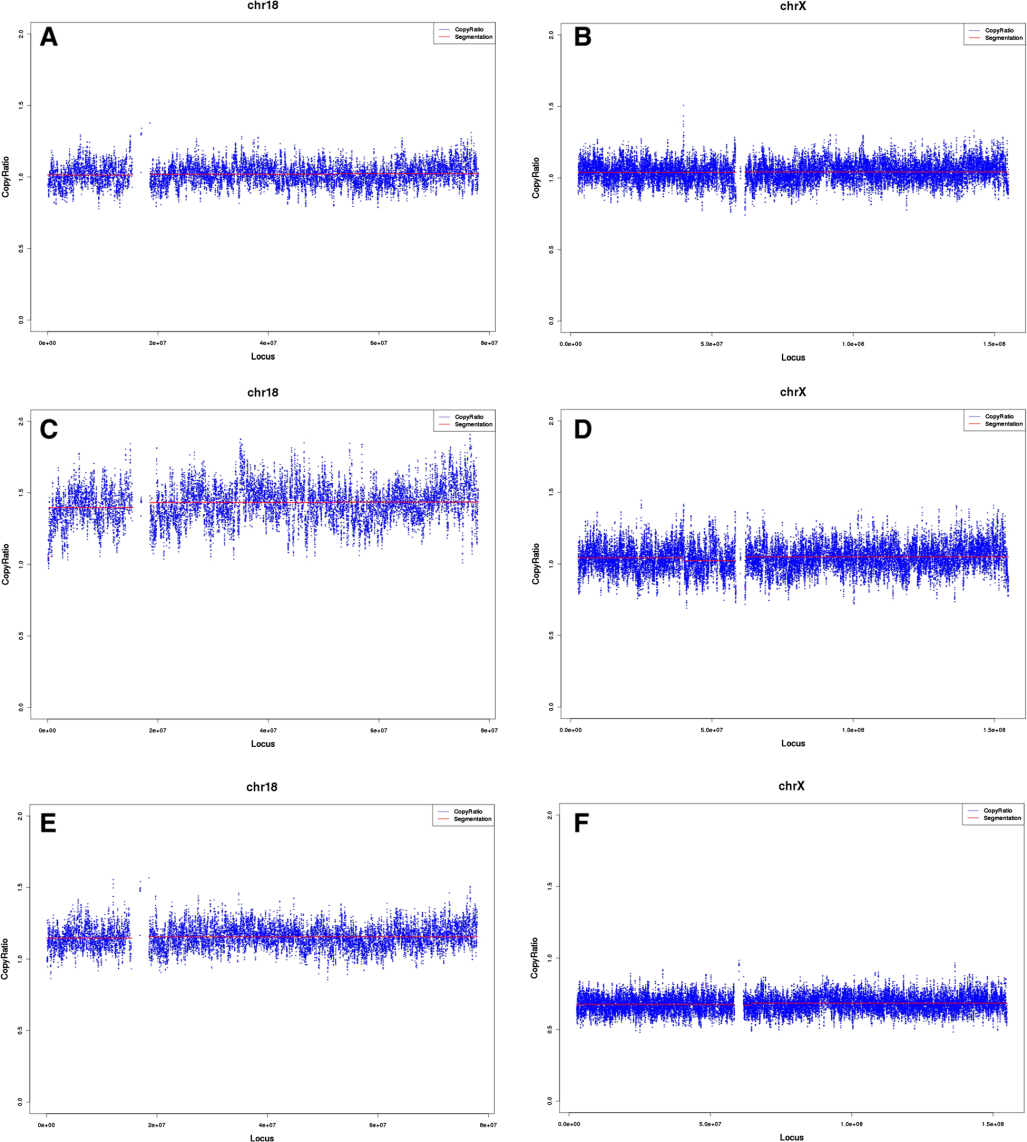
**The MPS results of maternal peripheral blood DNA, fetal DNA, placental DNA. A**-**B**. The maternal information of chromosome 18 and X, based on the sequencing result of maternal peripheral blood DNA, suggests a euploid status of the maternal background. **C**-**D**. The sequencing results of fetal DNA, where the copy ratio (1.5) of chromosome 18 in C indicated the trisomy 18 status in the fetus, the copy ratio (1.0) of chromosome X in D indicated the euploid status of chromosome X in the fetus. **E**-**F**. The sequencing results of the placental DNA, where the copy ratio (1.0-1.5) of chromosome 18 in E indicated a low level of mosacism of trisomy 18, the copy ratio (0.5-1.0) in F indicated a low level of mosaicism of monosomy X.

**Figure 4:**
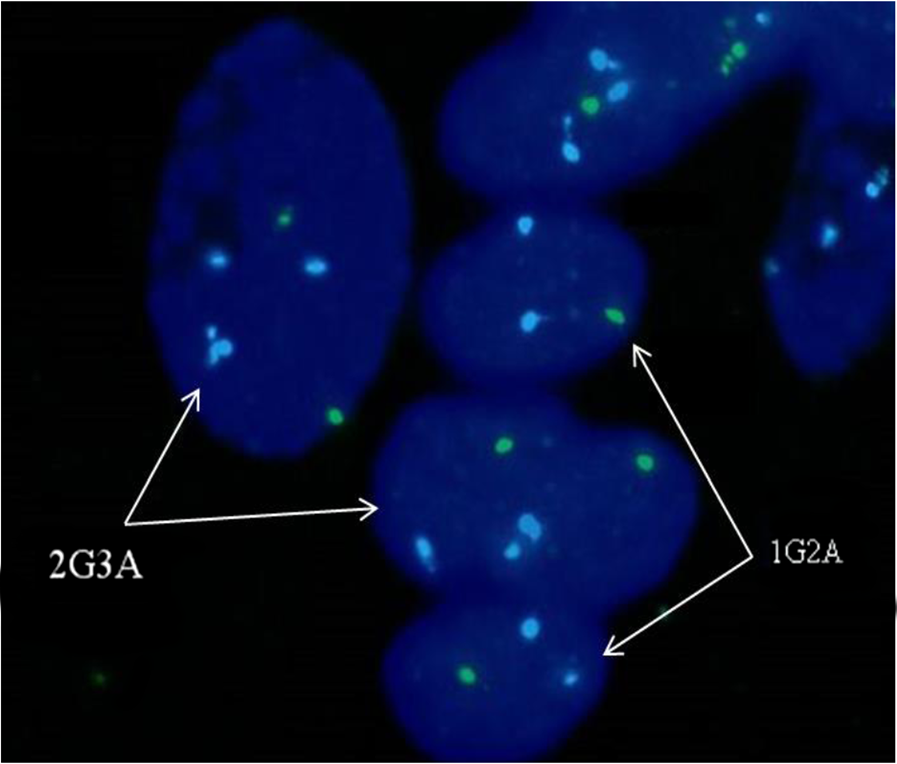
**FISH analysis results of placental tissue showed the combination status of 45, X and trisomy 18 mosaicism, with DNA of chromosome 18 marked as aqua (A) and chromosome X as green (G).** The cells with karyotype 47, XX, +18 were indicated as 2G3A, while the 45, X cells were indicated as 1G2A. Two additional chromatin segments attached to a chromosome 18 were observed in a cell (2G3A) on the left, suggesting partial duplications in addition to trisomy 18.

**Figure 5:**
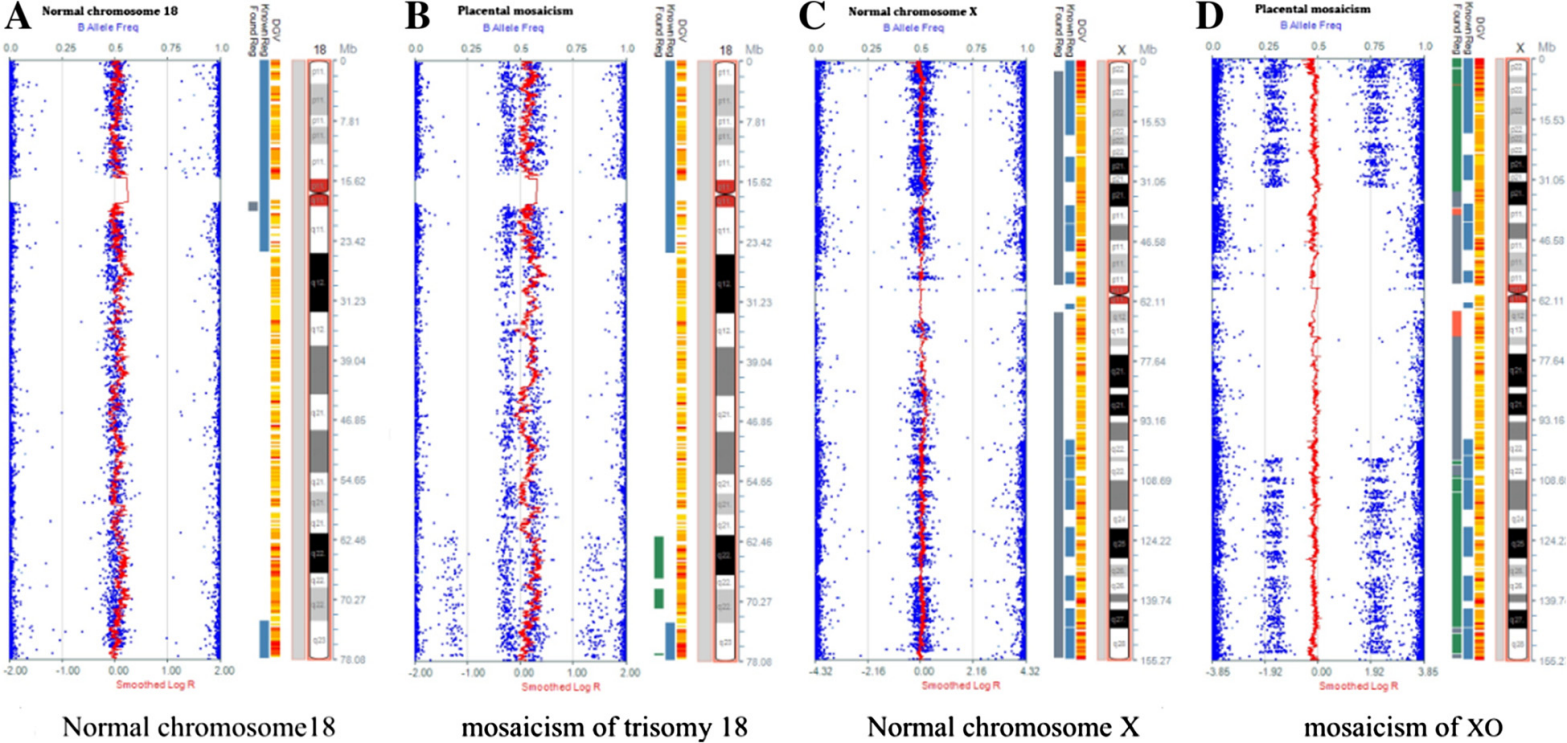
**Illumina SNP array result showing the presence of mosaicism of placental tissue. A**. A view of normal chromosome 18 as control of SNP array (Illumina HumanCytoSNP-12). The upper axis indicates the value of B allele frequency (BAF, blue dots). BAF may have three values (1 for BB, 0.5 for AB and 0 for AA) in a diploid individual. Additionally, the lower axis indicates the log*R* ratio (red line), where *R* illustrates the ratio of each SNP compared to diploid individuals in chromosome 18 (a log*R* ratio of 0 means an actual ratio of 1). **B**. A SNP array of placental tissue with mosaic trisomy 18. The BAF values (1 for BBB and BB; 0.6 for ABB and AB; 0.4 for AAB and AB; and 0 for AAA and AA) and the increased log*R* ratio indicated a mosaicism. Partial duplication in distal region of 18q was also observed. **C**. A view of normal chromosome X as control of SNP array (Illumina HumanCytoSNP-12). **D**. A SNP array of placental tissue with mosaic monosomy X. The BAF values (1 for BB; 0.75 for BB and BO; 0.25 for AA and AO; 0 for AA) and the decreased log*R* ratio indicated a mosaicism.

However, the NIPT did not reveal risks of fetal trisomy 13, 18 or 21, but demonstrated a great probability of 45, X. This was a female fetus. The t-score for chromosome 18 was -0.52, but for chromosome X was about -4.05, which suggests that the NIPT result was 45, X. Ultrasound scan at 19^+3^ week showed strawberry-shaped head and ventricular septal defect (VSD) (Figure 1). After consulting, the patient agreed to take a further amniocentesis for confirmation of NIPT results. G-banding of amniocytes cultivation identified the fetal karyotyping result as 47, XX, +18 (Figure 2) and implied that the NIPT had given a false negative result of T18. After post-test consultation, the couple decided to terminate pregnancy and agreed on additional research. Sequencing results of NIPT (Figure 3) identified the aborted fetal tissue as T18 condition, while the placental tissue as a status of mosaic T18 (mosaic ratio nearly 30%) and 45, X (mosaic ratio nearly 60%), which was in agreement with the amniocentesis result. Moreover, FISH analyses using chromosome 18 and X specific probes on placental tissues identified a combination of 45, X and T18 mosaicism, with high level of 45, X cells (67%, 62/92) and relatively low abundance of T18 cells (30%, 28/92) in total (Figure 4). This is also confirmed by SNP-array results of placental samples which offered clear evidence for the various level of 45, X and T18 mosaicism. The SNP-array results (Figure 5) with the signal intensity (logR) and genotyping (B allele frequency, BAF) value plots indicated the mosaic rearrangements. The increased logR and altered BAF value (Figure 5B) suggested a mosaic trisomy of chromosome 18 in 50% of the cells. The level of mosaicism was determined based on both logR and BAF value [3]. Notably, the result indicated partial duplication for the distal section of 18q represented in 17% of the cells, which was evidenced by the observation of two additional chromatin segments attached to a chromosome 18 in the FISH results (Figure 4). On the other hand, large mosaic deletion ranging in size from 31.05 to 93.16 Mb of chromosome X was illustrated in Figure 5C. The decreased logR and abnormal heterozygous BAF value indicated a mosaic monosomy at the level of 67%, which showed good agreement with the FISH results.

To the contrary, G-banding, FISH and SNP-array analyses on the post-mortem fetal tissue all demonstrated positive results of complete T18, showing that the fetal tissue is genetically different from the placental tissue.

## Analysis and conclusion

It is acknowledged that fetal cfDNA in maternal peripheral blood is originated from trophoblast and mainly consists of placental DNA [4,5]. However, the genetic discordance between placental and fetal tissue may affect the NIPT test and lead to inaccurate results. False positive NIPT results, small but not negligible, have been reported and noted for concern in these years [6-8]. In addition, mosaic condition of placenta may reduce the measurement accuracy and cause false negative result. The present case offered strong evidence for the idea and was supported by a previous case of double trisomy in placenta [9]. In this case, the fraction of fetal cfDNA for T18 caused by mosaic placenta is considerably lower than the detection threshold of NIPT test and consequently caused false negative results. Therefore, the level of mosaicism is an important factor for the NIPT test.

Collectively, considering the effect from placenta, the NIPT results should be interpreted combining other clinical tests under comprehensive background information.

## Consent

Informed consent was obtained from the patient for publication of this case report and any accompanying images. The sample collection procedures are approved by Huai’an Maternal and Child Health Care Hospital Medical Ethics Committee.

## Abbreviations

NIPT: Non-invasive prenatal testing; MPS: Massively parallel sequencing; CPM: Confined placental mosaicism; FISH: Fluorescence in situ hybridization; cfDNA: Cell-free DNA.

## Competing interests

The authors declare that they have no competing interests.

## Authors’ contributions

PQ carried out the molecular genetic studies and drafted the manuscript. SBJ, HXL and YHN reviewed all the clinical data and genetic counseling. JX, JFM, ZJ, LMM did the molecular cytogenetic analysis. HL helped to draft the manuscript, HP supervised the design of the SNP-array. NY supervised the laboratory work and helped to draft the manuscript. All authors read and approved the final manuscript.
